# Genome Mining and Comparative Analysis of *Streptococcus intermedius* Causing Brain Abscess in a Child

**DOI:** 10.3390/pathogens8010022

**Published:** 2019-02-13

**Authors:** Elio Issa, Tamara Salloum, Balig Panossian, David Ayoub, Edmond Abboud, Sima Tokajian

**Affiliations:** 1 Department of Natural Sciences, School of Arts and Sciences, Lebanese American University, Byblos 36, Lebanon; elio.issa@lau.edu (E.I.); tamara.salloum@lau.edu (T.S.); balig.panossian@lau.edu (B.P.); 2 Department of Neurosurgery, the Middle East Institute of Health University Hospital, Beirut 60-387, Lebanon; drdavidayoub@gmail.com; 3 Laboratory Department, the Middle East Institute of Health University Hospital, Beirut 60-387, Lebanon; abbouded@gmail.com

**Keywords:** *S. intermedius*, whole-genome, T7SS, GIs, VFs, SNPs

## Abstract

*Streptococcus intermedius* (SI) is associated with prolonged hospitalization and low survival rates. The genetic mechanisms involved in brain abscess development and genome evolution in comparison to other members of the *Streptococcus anginosus* group are understudied. We performed a whole-genome comparative analysis of an SI isolate, LAU_SINT, associated with brain abscess following sinusitis with all SI genomes in addition to *S. constellatus* and *S. anginosus*. Selective pressure on virulence factors, phages, pan-genome evolution and single-nucleotide polymorphism analysis were assessed. The structural details of the type seven secretion system (T7SS) was elucidated and compared with different organisms. *ily* and *nanA* were both abundant and conserved. Nisin resistance determinants were found in 47% of the isolates. Pan-genome and SNPs-based analysis didn’t reveal significant geo-patterns. Our results showed that two SC isolates were misidentified as SI. We propose the presence of four T7SS modules (I–IV) located on various genomic islands. We detected a variety of factors linked to metal ions binding on the GIs carrying T7SS. This is the first detailed report characterizing the T7SS and its link to nisin resistance and metal ions binding in SI. These and yet uncharacterized T7SS transmembrane proteins merit further studies and could represent potential therapeutic targets.

## 1. Introduction

*Streptococcus intermedius* (SI), is a facultatively anaerobic [1], microaerophilic, Gram-positive and beta-hemolytic organism [2], normally inhabiting the flora of oral, oropharyngeal and gastrointestinal tracts [3]. Along with *S. anginosus* (SA) and *S. constellatus* (SC), SI is a member of the *Streptococcus anginosus* Group (SAG). All are characterized by secreting proteolytic enzymes that damage tissues and contribute to abscess formation [4]. Within this family, SI is the most pathogenic as manifested by low survival rates and prolonged hospitalization [5]. Suppurative infections associated with pyrogenic exotoxin production by the SAG family are mainly described in the oral cavity and upper body regions [6]. However, the propensity is higher for SI to cause severe cases of brain and liver abscesses [7], most commonly after teeth manipulation [8]. The mechanism involved in cerebral infection is similar to that of *S. pneumoniae* [9]. Evasion by SI is also affected by other risk factors such as sinusitis, cyanotic congenital heart disease, otitis media, and dental caries [10].

The severity of SI associated infections is aggravated by the secretion of the human specific intermedilysin (ILY) [6]. Being a cholesterol-dependent cytolysin, ILY binds to the human complement regulator CD59 (hCD59) and as a result is a main virulence factor (VF) in SI [11]. The regulation of ILY production is accomplished by the catabolite control protein A based on the accessibility to nutrients [12]. Moreover, invasion of human cells by SI is possible and controlled by the secretion of a hydrolytic enzyme, hyaluronidase, which facilitates biofilm formation [13]. Pathogenicity is also increased through dynamic proliferation linked to sialidase activity [14], the polysaccharide capsule [15], and expressing antigenic properties such as surface proteins. Surface proteins, such as antigens I/II that bind to proteins of the extracellular matrix, fibronectin, and laminin [16], promote an inflammatory response [17]. 

With scarce patient data and only 16 genomes identified as SI being available on NCBI, seven of which are complete genomes, the genetic components underlying pathogenicity, the mechanisms involved in brain abscesses development, and genome diversity are not well characterized. In this study, we performed a whole-genome characterization and comparative analysis of LAU_SINT, an SI isolate recovered and linked to brain abscess formation in a 13-year-old boy. We also studied the structural details of the type seven secretion system (T7SS) in SI and related organisms and based on that proposed the presence of four T7SS modules (I–IV) located on various genomic islands (GIs).

### Case Presentation

A 13-year-old patient was admitted to the Middle East Institute of Health University Hospital (MEIH) on January 2018 presenting several episodes of helmet headaches decompensated by a resolution of the state of consciousness with an epileptic seizure of grand mal type. Upon admission, blood test results performed on 20 January 2018 revealed signs of an infection characterized by leukocytosis (18,800/mcl) with 81% neutrophils and high C-reactive protein levels (CRP = 427.5 mg/L).

**Figure 1:**
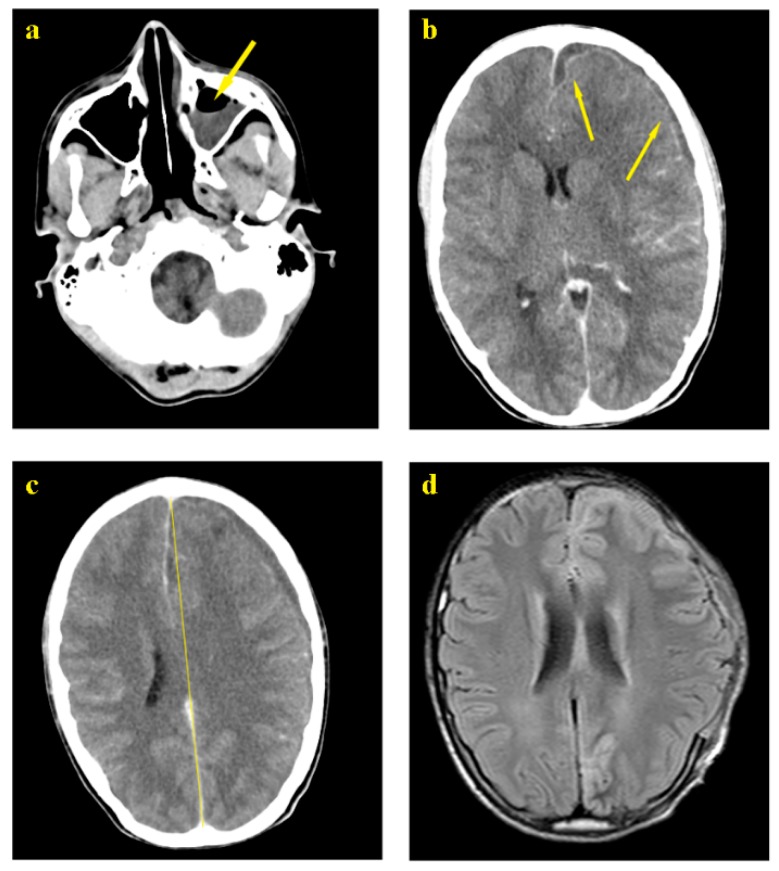
(**a**) Left ethmoid sinusitis; (**b**) Meningitis; (**c**) Meningitis and midline shift front to parietal collection; (**d**) Brain MRI post decompression (craniotomy).

A non-injected brain CT scan on 22 January 2018 revealed the presence of sinusitis and a left holoencephalic millimeter hypodensity attributed, according to the treating specialist, to a thin blade of chronic subdural hematoma with his recommendations to treat it medically and conservatively (Figure 1a).

Despite the antibiotherapy with 1 g of Ceftriaxone IV twice a day through IV, the patient continued to deteriorate neurologically, with crises that became more frequent, a complete resolution of the state of consciousness imposing the need to intubate. A second scan revealed the stability of the subdural collection, but with a huge increase in the underlying edema that extended into the white matter but did not look like a glove. Moreover, a deviation of the median line of more than 1 cm was reported (Figure 1b). 

A secondary diagnosis suggested the presence of empyema rather than hematoma. This hypothesis was ignored and conservative treatment was continued until bilateral mydriasis appeared along with abolition of reflex brainstem urging the necessity of a surgical procedure. A decompressive cranectomy with an enlargement duroplasty was performed and revealed a very abundant empyema consisting of encysted thick pus molding the cortical surface, infusing through the cortical sulcus and presenting multiple septa. With no significant complications, the surgery resulted in a complete resolution of mydriasis and restoration of the normal brain color and beating rhythm. No signs of meningitis were further identified in the brain MRI post decompression on 8 February 2018 (Figure 1c,d).

The culture showed growth of a Gram-positive cocci in chains and colonies appeared gamma haemolytic on blood agar. The isolate was catalase negative. It did not agglutinate any Lancefield antigen sera, didn’t grow on bile esculin agar or 6.5% NaCl broth. The Strep API system (bioMerieux, La Balme les Grottes, France) was inconclusive. The result was issued as Viridans *Streptococci* and was further identified using 16S rRNA sequencing as SI.

## 2. Results

### 2.1. Genomic Features

The LAU_SINT draft genome sequence consisted of 1,950,264 bp with 37.7% G+C content distributed in 36 contigs. Annotation of this assembly using RAST identified 1898 coding sequences (CDS), 50 tRNAs, and four rRNAs. Genes linked to carbohydrates (239), protein metabolism (204), amino acids and derivatives (140), and cell wall and capsule (120) were the most abundant among the SEED subsystem categories. 

All 17 NCBI available genomes (accessed on 25 October 2018) listed as SI were included in this study (Table 1). Our results showed that 567_SINT and F0395 listed as SI on NCBI, belonged to SC. We additionally confirmed this through chromosomal comparison and whole-genome based single nucleotide polymorphism (wgSNPs) analysis where 567_SINT and F0395 clustered with SC (Figure S1).

The average genome size of the 15 SI genomes was 1,953,043 ± 42,147 bp. The G+C content was highly similar ranging from 37.5% to 37.7%. The isolates had on average 1926 ± 55.8 CDS.

### 2.2. Resistance Profile

LAU_SINT was susceptible to the 12 tested antibiotics (Table S1). We couldn’t detect any acquired resistance determinant using ResFinder 3.0 [18]. LAU_SINT carried the *S. pneumoniae* vancomycin tolerance *ex123*-*pep27*-*vncRS* locus, a multidrug resistance efflux pump PmrA, multi-antimicrobial extrusion protein (Na(+)/drug antiporter), and MATE family of MDR efflux pumps. Nisin immunity protein, conferring resistance to nisin, was detected in LAU_SINT and seven of the isolates retrieved from NCBI (47.1%) (30309, 32811, B196, C270, F0413, TYG1620, and 631_SCON). 

### 2.3. cg-SNPs Phylogeny and Recombination

**Figure 2:**
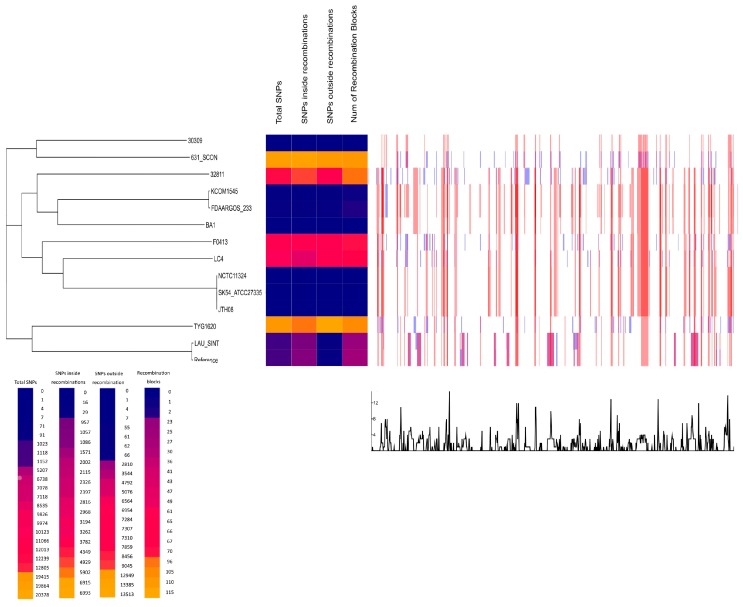
Core genome (cgSNP)-based phylogenetic tree and genome-wide recombination hotspots.

The core genome SNPs based phylogenetic analysis was based on 1,996,214 nucleotides common in the studied isolates and the reference complete genome B196 (CP003857.1). All samples were 82–87% aligned with the reference base pairs, except our isolate LAU_SINT that mapped with 92.33% alignment to the reference and clustered closest with it in the phylogenetic tree (Figure 2). 

Clear-cut regions were observed in favor of recombination, with a few isolates sharing of genetic shuffling. TYG1620 was overly distinct in its high total number of SNPs, both inside and outside the boundaries of recombination blocks. F0413 and LC4 also clustered close to each other, having similar preference in recombining loci.

It is noteworthy, that a 16S rRNA based phylogenetic clustering had a lower discriminatory power for speciation compared to cgSNPs-based phylogeny (Figure S3). 

### 2.4. dN/dS Ratio

In order to infer the presence of evolutionary selective pressure on the protein-coding CDS genes in SI, the dN/dS ratio was calculated based on the Tajima’s D method [19]. B196 was used as a template for calculating the dN/dS ratio in SI. The average dN/dS ratio was 0.170 ± 0.193 for all the isolates having both dN > 0 and dS > 0. Since the average ratio was significantly less than one, a negative selection is suggested. Three regions in LAU_SINT had a dN/dS ratio > 1 ranging from 1.08 to 1.3, suggesting diversifying selection (Table S2). The first region (185,065 bp to 185,122 bp) was one of a group of several hypothetical proteins, and the second region with dN/dS = 1.17 (1,995,445 bp to 1,996,180 bp) was a chromosome-partitioning protein Spo0J with a diversifying selection in all the isolates except SK54. The third region (348,809 bp to 349,301 bp) encoded a GNAT family acetyltransferase and showed a diversifying selection in all tested isolates.

### 2.5. Phage Content

In total, 14 different phages were identified among the genomes ranging between one to four per isolate. *Bacillus* phage G (accession # NC_023719) was the most common (52.9%; *n* = 9) followed by *Streptococcus* phage phiARI0131-1 (accession # NC_031901) (29.4%; *n* = 5). The majority of the detected phages were incomplete except for *Lactobacillus* phage PLE2 (accession # NC_031036) in 567_SINT, *Campylobacter* phage PC14 (accession # NC_031909) in F0395, and *Streptococcus* phage phiARI0468-1 (accession # NC_031929) in LAU_SINT. Phages covered more than 4.5% of the genomes (Figure S4). 

Alignment of LAU_SINT and B196 revealed that the major differences between the two genomes resided in the phage DNA content being detected in LAU_SINT and missing from B196 genome; LAU_SINT carried one additional intact *Streptococcus* phage phiARI0468-1 (accession # NC_031929) not present in B196.

### 2.6. Virulence Factors

The virulome of the isolates was determined through BLAST search on RAST. A common set of VFs was observed among the 17 isolates except for 567_SINT and F0395. Adhesion in SI was linked to five genes (*eno*, *fbp54*, *psaA*, *lmb*, and *pulA*), which were detected in all the isolates. Invasive properties were associated with a complete *sil* locus (*sil*A-E), capsular proteins (*cps*19FL-O), and *gal*U coding for UTP-glucose-1-phosphate uridylyltransferase. Metabolism, invasion, and colonization were evenly encoded in all the 17 isolates and were associated with *csrR*, *salX*, and *gapDH* genes, respectively.

Also, *ily* and *nan*A encoding intermedilysin and sialidase, respectively, were found in all the isolates except for 567_SINT and F0395. The dN/dS for both *ily* and *nanA* ratio was <1 suggesting a negative purifying selection. 

Nine of the 15 SI isolates carried a homologous gene (*inlA*) for internalin A of *Listeria monocytogenes* linked to invasion [20]. Hemolysin III was detected in all SI isolates. 

### 2.7. Type VII Secretion System

We performed a comparative genomic analysis of the T7SS in all the sequenced isolates including LAU_SINT. Based on that we came up with four different modules representing the T7SS in SI designated as module I to module IV. All the genetic elements coding for the T7SS in all the sequenced isolates were detected on GIs, with all additionally having other elements not directly linked to the T7SS (Table S3).

Module I was identified in six SI isolates (40%) (TYG1620, 32811, BA1, F0413, FDAARGOS_233 and KCOM_1545), integrated between *adhE* (bifunctional aldehyde/ alcohol dehydrogenase) and *nanE* (N-acetylmannosamine-6-phosphate 2-epimerase), and differed by the number of hypothetical proteins present between *essC* and *esaA* (ranging from 6 to 15 proteins). Module I was detected on a 42 Kb GI with a G+C content of 35.3%, indicating its acquisition through horizontal gene transfer (HGT). It also had a different genetic arrangement than what was previously described with the 21 kb GI carrying a T7SS in *S. suis* serotype 9 strain GZ0565 [21], and from a pathogenicity island carrying WXG100 secretion system in *S. agalactiae* [22].

Module II was also detected in six other SI isolates (40%) (30309, JTH08, LC4, NCTC11324, SK54, and 631_SCON). Module II was located on a 17.3 Kb GI with a G+C content of 35.6%, significantly different from Module I and from the ones detected in *S. suis* and *S. agalactiae* [21,22].

Module III was found in two isolates (13%) (B196 and C270) with little similarity to Modules I and II. This Module was located on a predicted 23.6 Kb GI with a 36.3% G+C content, and was not predicted in silico using online tools.

Module IV was detected in LAU_SINT and in only other two SC isolates (567_SINT and 783_SANG). The T7SS in the SC genomes was located on a 40.6 Kb GI with a 37.8% G+C content, while it was on a 63.6 Kb GI in LAU_SINT with a G+C content of 38.8%. In LAU_SINT, we additionally detected on the same GI the nisin immunity protein. Module IV had similar genetic arrangements to the GI carrying T7SS in *S. agalactaiae* [22].

No T7SSs were detected in the SA genomes indicating a more recent acquisition in SI through the HGT of GIs. 

Of interest, Modules I, II and IV also had additionally genes linked to metal ions binding such as bifunctional acetaldehyde-CoA/alcohol dehydrogenase, VWA domain-containing proteins, magnesium and cobalt transporter, and ABC transporters. 

Comparative analysis of the four T7SS modules showed a uniform distribution of the core *essC*, *essB*, *esaA* and *esxA* T7SS genes. Modules I and II carried additional *essA* and *esaC* genes while modules II and IV had extra *esaB* and *essD* genes, respectively. Modules I and III had an LXG domain-containing gene in common.

Based on the suggested T7SS module classification, each isolate was compared to the module reference for presence/absence of genes within the T7SS locus. In Module I, genes showed on average 7.27% variability. Genes coding for a cell wall surface anchor family protein and a DUF11 domain-containing protein were missing in 60% of Module I isolates (TYG1620, BA1, and KCOM 1545). F0413 lacked an isopeptide-forming domain-containing fimbrial protein and a DNA repair protein. Three isolates (32811, BA1, and KCOM 1545) displayed two to four different hypothetical proteins (HP) than the reference. In Module II, LC4 and 631_SCON had three missing HP (60% of HP). In Module III, B196 and the reference C270 showed a 94.12% HP similarity with only 1 HP absent in B196.

**Figure 3:**
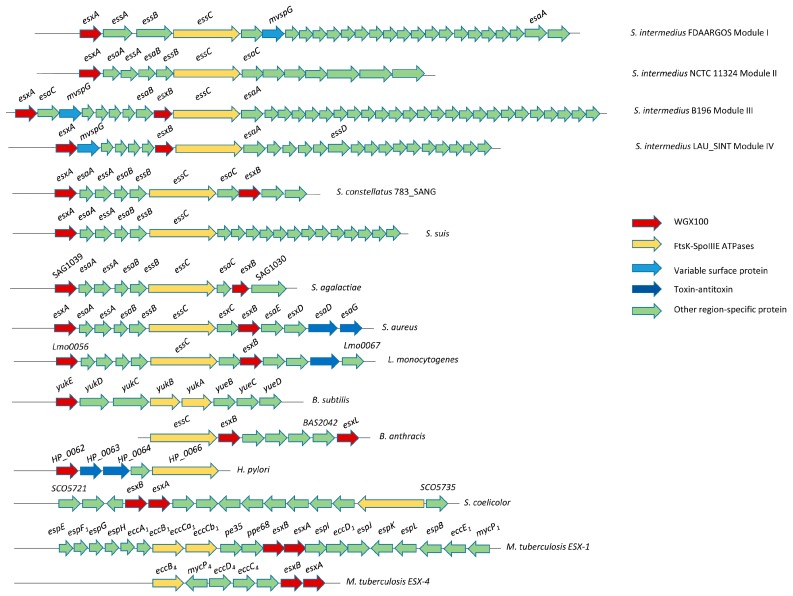
Schematic representation of the genetic loci T7SS (or T7SS-like) in SI in comparison with other organisms.

We also compared the four T7SS Modules in SI and SC to that in *Mycobacterium tuberculosis* ESX-1 and ESX-2 systems, *Staphylococcus aureus* strain USA300, *Bacillus subtilis* strain 168, *Bacillus anthracis*, *S. agalacticae* 2603V/R, *S. suis*, *Streptomyces coelicolor* A3(2), *L. monocytogenes*, and *Helicobacter pylori* strain 26695. The T7SS and T7SS-like were highly diverse among the different organisms. EsxB substrate was absent in all SI except in LAU_SINT. Module I was negative for EsaB and EsaC regulatory proteins, which were detected in *S. aureus* and *S. agalactiae*. In all cases, *esxA* forming the WXG100 motif was located in gene clusters that also had a conserved ATPase (*essC*) and several FtsK/SpoIIIE domains (Figure 3). 

**Figure 4:**
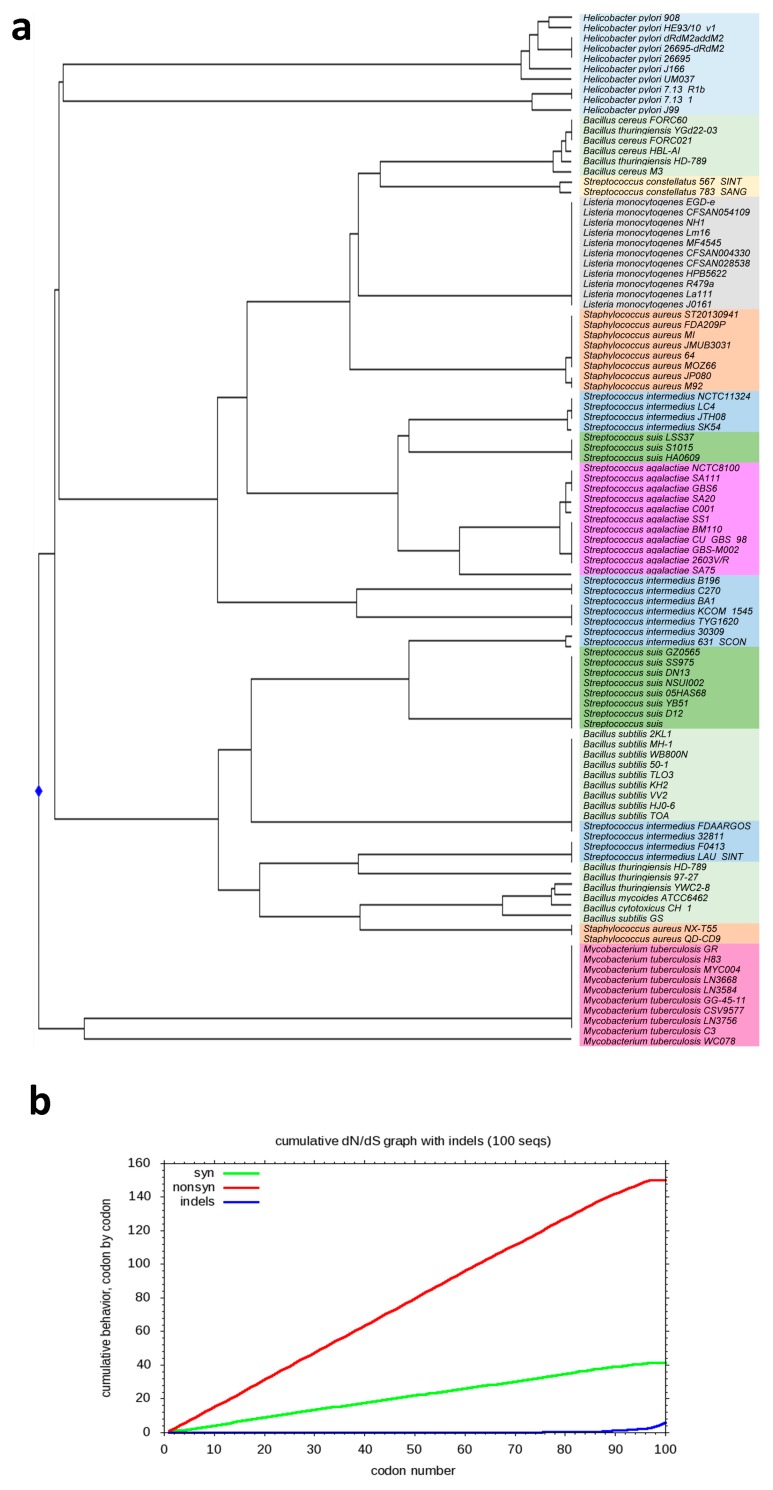
(**a**) UPGMA (unweighted pair group method with arithmetic mean) phylogenetic tree of *esxA*; (**b**) Cumulative dN/dS ratio of *esxA*. Syn: synonymous mutations; nonsyn: non-synonymous mutations; indels: insertions and deletions.

*esxA* was retrieved from a variety of organisms carrying T7SS or T7SS-like. A total of 100 entries were used to build a UPGMA phylogenetic tree. *esxA* appeared to be conserved within the same species except in SI, *Bacillus*, and *S. aureus*. *esxA* in SI was most closely related to *S. suis* and was conserved in *S. suis* within two separate lines of descent. This could indicate a possible exchange of the T7SS elements through HGT between the two isolates. *esxA* in SC was more distantly related. The average dN/dS for pairwise comparisons of *esxA* was 1.102, indicating a positive evolutionary selection (Figure 4). 

### 2.8. Comparative Genome Analysis 

**Figure 5:**
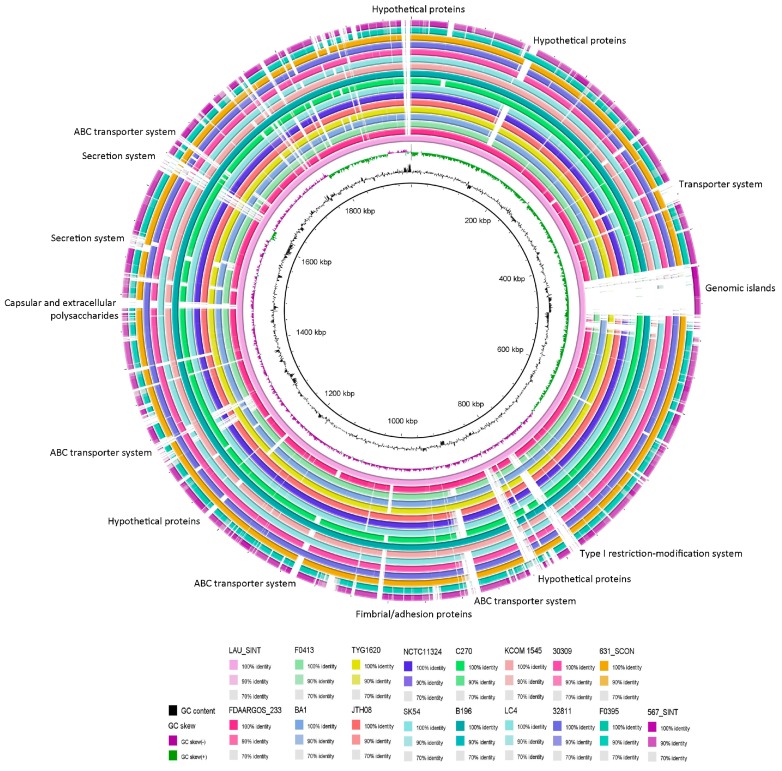
Circular genomic map and genome comparison of the 17 genomes. The circle is divided into arcs representing the genomes as labeled. The black histogram represents the G+C content and purple-green histogram represents the G+C deviation. The function of genes which were part of major deletion events are labelled at the edge of the rings.

Full chromosome comparison revealed similarity rates between the SI genomes ranging from 52% to 100%, with JTH08 and NCTC 11324 being 100% similar. A comparative analysis between LAU_SINT and B196 on RAST revealed that only 78 genes were unique to LAU_SINT while 101 genes were unique to B196. The uniquely identified genes in LAU_SINT included hypothetical proteins, phage proteins, and a metallo-β-lactamase family protein (matching a metallo-hydrolase in *Staphylococcus xylosus* PNZ15953). The unique genes in B196, however, included hypothetical and CRISPR-associated proteins. Circular genome comparison of the 17 isolates, used to examine major deletion events, revealed the presence of a large phage in LAU_SINT and 567_SINT matching that of *Clostridium* phiCT453B (accession # NC_029004). Variability between the genomes was mainly due to differences in secretion and transporter systems and GIs (Figure 5).

All SC (*n* = 13) and SA (*n* = 46) genomes available on NCBI, as of 20 October 2018, were retrieved and used for full chromosomal comparison and the construction of an UPGMA wgSNPs-based phylogeny. Based on that we concluded that SI was more closely related to SC and have more recently evolved from SA, with 567_SINT and F0395 clustering with other SC genomes (Figure S1). The dN/dS ratio was additionally determined for SAG. In total, 176 regions had a dN/dS ratios > 1 (ranging from 1.01 to 4.5), 13 of which were detected in LAU_SINT (Table S2). 

### 2.9. Pan-Genome Analysis

**Figure 6:**
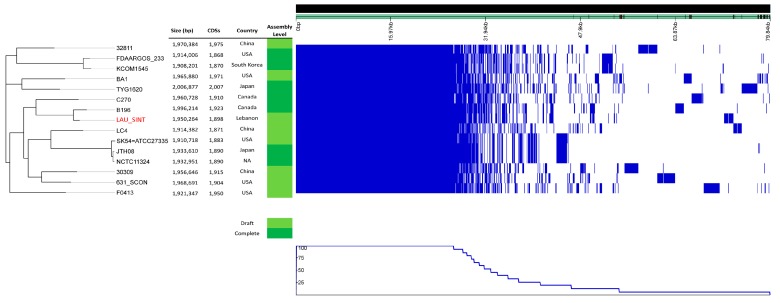
Visualization of a maximum-likelihood phylogenetic tree, genome statistics, and assembly level of SI pan-genome.

The pan-genome in the 17 isolates was first determined using Roary^23^ with the cut-off for BLAST hits being set at 95% and core gene prevalence at >99%. Isolates 567_SINT and F0395 had distinct pan-genome distribution and as a result were excluded from subsequent analysis (Figure S5) to only include SI isolates (Figure 6). A total of 3990 genes were included. The core genome (≥99% of isolates) was estimated to consist of 1327 genes. The analysis of differentially present/absent genes revealed 991 shell genes present in two or more isolates (15% ≤ isolates < 95%) and 1672 unique cloud genes specific to single isolates (0% ≤ isolates < 15%). 

Shell and cloud genes, constituting the accessory genome, were thoroughly examined. 1872 genes encoded hypothetical proteins. These were removed from subsequent analysis. Duplicates encoding the same protein product were manually inspected and removed. Accordingly, 325 genes that were present in 14 or less of the 15 SI isolates were retained. Genes related to drug resistance, toxins and transport systems were depicted in Figure S6. 

## 3. Discussion

Despite being the least commonly isolated species in the SAG, SI is often associated with a significantly longer hospital stay and a higher 30-day mortality in infected patients [5]. With the scarcity of whole-genome data available and the lack of infection-associated metadata, it is of great importance to perform whole-genome based characterization of symptom-specific SI isolates in an attempt to elucidate the molecular basis of pathogenesis. In this study, we performed a comparative genome analysis on LAU_SINT, linked to a brain abscess in a 13-year-old boy admitted to a tertiary hospital in Lebanon, with all publicly available SI genomes and other members of SAG. SI genomes were conserved with variations in the phage contents, secretion and transport systems. *ily* and *nanA* were both abundant and conserved. We proposed based on the results of our study four T7SS modules (I–IV), which were located on various genomic islands and showing similarity to that of *S. suis* and *S. agalactiae*. 

All 17 NCBI-available genomes listed as SI were included in this study. Subsequent analysis showed that isolates 567_SINT and F0395 belonged to SC. The identity of isolate F0395 was previously shown by Jensen et al. (2013) [1]. The SI isolates were deposited on NCBI between 2011 and 2018 and were collected from USA, Canada, China, South Korea, Japan, and recently Lebanon (LAU_SINT). Many isolates had missing clinical and patients’ data. The SI genomes had an average size of 1,947,393 bp ± 31,259 bp and a conserved pan-genome consisting of 1327 core genes compared to 626 genes in the genus *Streptococcus* and 1234 genes in the SAG [23]. cgSNPs analysis provided enough discriminatory power to separate the SI from 567_SINT and F0395 SC isolates but did not infer symptoms- or geo-based distribution [23]. The calculated dN/dS based on the Tajima’s D method [19] suggested a strong purifying or stabilizing selection in all the SI isolates. Diversifying selection was observed in a GNAT family acetyltransferase that was previously shown to be over-expressed in abscesses [9]. Differences between the genomes were mainly attributed to transporter and secretion systems perhaps used to manipulate the host, establish a replicative niche, or compete with nearby microorganisms [24]. 

The isolates carried a plethora of VFs associated with adhesion, regulation of metabolic activities, invasion and colonization, and capsule-linked proteins. Colonization and adhesion could be initiated through the expression of the *fbp*54 and *lmb* coding for fibronectin and laminin binding proteins (Antigens I/II), respectively [25]. Binding was found to activate an immune response causing cytokine buildup followed by tissue damage and onset of brain abscess formation [17]. Other adhesion proteins were also detected such as surface protein (*psa*A) [26], enolase (*eno*) for plasminogen binding, and pullulanase (*pul*A) [27]. The invasive properties of the 17 isolates were associated with a complete *Streptococcus* Invasion locus (*sil*) system and an internalin A homologous gene (*inlA*). The *sil* locus enhanced virulence in the SAG group [28] and allowed for competitive microbial interactions with host bacteria [29]. On another note, all SI isolates displayed homologues for the polysaccharide capsule operon of *S. pneumoniae* (*cps*4 A, B, C, D, and E) [30], with the *cps*4E being absent in both 567_SINT and F0395. Hemolysis of red blood cells in SI relies on having hemolysin III. That is opposed to the virulent *S. agalactiae* hemolysin proteins encoded by *cyl*A, G, and Z [31]. The presence of *luxS*, *nanA* and *ily* is alarming since the outcome of the infection is worsened by their ability to produce biofilms regulated by *lux*S and its product, autoinducer-2 [32]. Further tissue damage is caused by sialidase production encoded by *nan*A, while intermedilysin, the product of *ily*, is the major VF linked to pathogenicity [6]. *ily* and *nanA* are under purifying selection, a phenomenon associated with slow rates of evolution in essential genes [33]. *luxS* further controls the expression of *ily* and *hyl* which can be downregulated by a nonfunctional AI-2, translation product of a mutant *luxS* gene. Mutations in the *luxS* gene attenuate the hemolytic activity of *ily*, but with no significant effect on hyaluronidase activity and expression of *fbp54* and *lmb* [34]. In the absence of mutations, intermedilysin perforates membranes of human erythrocytes [35] and induces RBC programmed necrosis favoring microbial growth especially that of pore forming toxin producing hCD59-dependent pathogens [36] such as SI. Hyaluronidase, second major VF, acts as a pyogenic growth factor by degrading hyaluronan in host tissues [37] to provide bacterial nutrients and facilitate pus formation [38]. Tissues also become less viscous and more permeable, inducing bacterial mobilization [39]. 

We found that almost half of the SI isolates carried nisin immunity proteins conferring resistance to nisin; a bacteriocin commonly found in mouth washes and used against pathogenic bacteria related to dental caries and root canal infections such as *S. mutans*, *S. sanguinis*, *Lactobacillus acidophilus*, and *Enterococcus faecalis* [40]. As such, we believe that this resistance determinant could have favored SI survival and facilitated infections following dental manipulation as was also previously suggested with other organisms [8]. 

We identified a total of 14 different phages that accounted for more than 4.5% of the genomes. *Bacillus* phage G (accession # NC_023719) was the most common followed by *Streptococcus* phage phiARI0131-1 (accession # NC_031901). *Clostridium* phage was identified in LAU_SINT and carried a *pblB* related gene previously reported as being associated with endocarditis in *S. mitis* infections [41]. Despite the scarce amount of information on the role phages play in the evolution and pathogenicity of SI, our results also implied that the emergence of this species relied at least partially on HGT driven by the acquisition of the proteinic phage-encoded, hyaluronidase [41] and virulence inducing antigens I/II [26]. 

This study showed that 16 out of the 17 (94.1%) analysed SI genomes harboured a putative T7SS. Based on their genetic arrangement, we proposed and distributed them into four Modules carried by four different GIs. In six isolates, the T7SS, identified as belonging to Module I based on our suggested grouping, was present on a 42 Kb GI with a G+C content of 35.3% implicating its acquisition through HGT. This GI had a different genetic arrangement than a previously described 21 kb GI carrying a T7SS in *S. suis* serotype 9 strain GZ0565 yet absent in eight other SS2 virulent strains [22]. We identified 10–15 transmembrane proteins in this module that might contribute to the formation of the membrane pore [42]. The three other modules of the T7SS had a distinct genetic organization and were also linked to different GIs. These differences in gene content could contribute to the complexity and/or role played by the T7SS. Previously, genetic variability of the T7SS was addressed across *S. aureus* where four distinct modules were identified based on the variability observed within the *ess* loci. This variability was hypothesized to be driven by recombination events and suggested that it was more recently acquired in Firmicutes compared to T7SSs in mycolic-acid containing bacteria [43]. In fact, *M. tuberculosis* encodes five T7SSs, with ESX-1 and ESX-5 being linked to virulence. Following internalization into macrophages, ESX-1 secreted factors acted as pore forming toxins circumventing phagolysosomal degradation and facilitated translocation inside the host. ESX-5 secreted proteins manipulated immune response of macrophages inducing cell death and promoted bacterial dissemination [44].

Although one specific function could not be attributed to the diverse modules of the T7SS found in SI, the presence of genes linked to metal ions binding on the different GIs carrying T7SS suggested its possible involvement in the establishment of the infection and abscess formation. The roles of iron, manganese and zinc have been extensively studied in *S. aureus* [45] and *S. pneumoniae* infections [46] but not in SI pathogenesis. Thus, uncovering the pathways linked to metal acquisition in SI could help to find potential therapeutic targets. The T7SS further mediates interspecies competition through the secretion of three main LXG toxin proteins (TelA, B, and C) via its ESX pathway. TelC phosphatase targets the lipid II precursor of bacterial cell walls and destroys it, jeopardizing the survival of neighbouring bacterial species [47].

As shown in this study for SI and in other organisms, *esxA* forming the WXG100 motif was located in gene clusters that also encoded a conserved ATPase (*essC*) containing several FtsK/SpoIIIE domains and that was predicted to power T7SS [48]. The T7SS substrate, EsxA, was shown to contribute to *S. suis* virulence in a mouse infection model [22]. EsxA also delayed host cell apoptosis, and together with EsxB facilitated the release of intracellular *S. aureus* from human epithelial cells [49]. The distribution of WXG100 proteins was first thought to be restricted to Gram-positive phyla *Firmicutes* and *Actinobacteria* before being identified in *Cyanobacteria*, *Lentisphaerae*, *Proteobacteria* and *Verrucomicrobiae* [50]. A GI encoding a TerY-phosphorylation triad in addition to components of T7SS were found in *Helicobacter pylori* [51]. Despite having structural conservation in various organisms, the sequence identity among the WXG100 proteins was only about 15% [52]. *esxA* was under positive selective pressure and appeared to be evolutionarily related in SI and *S. suis* within two distinct lines of descent. 

The protein composition of the T7SS differed between our four suggested Modules. EssA, EssB, and EssC were conserved in SI compared to other organisms; required for transporting substrates EsxA and EsxB across the envelope [53]. However, similar to *S. suis*, Modules I and II of the T7SS in SI lacked EsxB compared to *S. agalactiae* [22]. Previously, *S. aureus* pan-genomes analysis showed a distinct set of genes downstream of the core *ess* genes, with most not having the EsxB, EsxC, EsxD, or EsaD substrates [53]. Also absent in Modules I and IV were EsaB and EsaC; EsaB is a cytoplasmic protein that appears to regulate the Ess machinery [53], acting as a negative regulator of EsaC. EsaC contributed to persistent *S. aureus* infections [53], which was also absent in *S. suis* [22]. The absence of the regulatory protein EsaB suggested that the T7SS in SI has other yet to be identified regulatory mechanisms and that the complete repertoire of Ess-secretion substrates and their corresponding functions in T7SSs are yet to be elucidated. 

In this study, the genome of an SI isolate, LAU_SINT, linked to a brain abscess of a 13-year old child following sinusitis was characterized through whole-genome based comparison with other SI and SAG genomes. High levels of genome conservation were observed despite the isolates being collected from various geographic regions and infection sites. VFs were conserved and widespread. Differences were attributed to changes in transporter, secretion systems, with GIs and HGT driving genome evolution. The role of the T7SS in SI virulence and the roles of the different proteins linked to metal binding and located on the same GIs as those of the T7SS merit further detailed studies. Finally, the correlation, if any, between the T7SS modules that were revealed in this study and SI virulence, pathogenesis, and abscess formation is also to be determined.

## 4. Materials and Methods 

### 4.1. Ethics Statement

Ethical approval for this study was obtained from the Institutional Review Board Committee of the Middle East Institute of Health, University Hospital with the following approval number: AL-01-03/2018-IRB/MEIH.

### 4.2. Antimicrobial Susceptibility Testing

Antimicrobial susceptibility testing was performed using the disk diffusion method against 12 antibiotics: Amoxicillin, amoxicillin-clavulanic acid, Gentamycin, kanamycin, Streptomycin, Levofloxacin, Erythromycin, Clindamycin, Tetracycline, Vancomycin, Bactrim, and Rifampicin. The obtained results were interpreted according to the EUCAST breakpoints as updated in 2015 (http://www.eucast.org) [54].

### 4.3. DNA Extraction

The bacterium was cultured on 5% Blood agar DNA under anaerobic conditions at 37 °C. DNA extraction was performed using the Nucleospin Kit (Macherey-Nagel, Düren, Germany) according to the manufacturer’s instructions.

### 4.4. 16 S rRNA Sequencing

The 16S rRNA gene was amplified using primers SSU-bact-27F (5′-AGAGTTTGATCMTGGCTGAG-3′) and SSU-bact-519R (5′-GWATTACCGCGGCKGCTG-3′) [55]. The resulting PCR products were sequenced on the Genetic Analyzer 3500 (Applied Biosystems™, Foster City, CA, USA). Obtained sequences were extracted using Sequencing Analysis v5.4 software (Applied Biosystems), assembled using CLC Main Workbench v7.0.2 (CLC-bio, Aarhus, Denmark), and blasted against the NCBI 16S rRNA database.

### 4.5. Genome Sequencing 

Genomic DNA (gDNA) was used as input for library preparation using the Illumina TruSeq DNA library preparation kit (Illumina, San Diego, CA, USA). The library was multiplexed, clustered, and sequenced on an Illumina MiSeq with paired-end 500 cycles protocol to read a length of 250 bp.

### 4.6. Genome Assembly and Annotation

Quality control was done using FastQC [56], reads were demultiplexed, and low quality base pairs were trimmed using trimgalore [57]. Genome assembly was performed de novo using SPAdes with default parameters [58]. The genome was subjected to BLAST analysis against Resfinder 3.0 [18]. PHASTER was used to detect putative phage sequences in the genome [59]. RAST was used to search for putative VFs [60]. IslandViewer 4 was used to predict possible GIs [61]. Genes were verified manually and blasted against the NCBI sequence database. Proteins were identified as cytoplasmic or membrane-embedded using Protter version 1.0 [62]. SNAP v2.1.1 was used to calculate synonymous and non-synonymous substitution rates [63].

### 4.7. cgSNPs Analysis

Core genome SNPs were called for each isolate using the Snippy pipeline [64], each isolate was mapped to the reference genome B196 (CP003857.1). RAxML v8 [65] was run with 1000 bootstrap iterations to generate a Maximum Likelihood (ML) phylogenetic tree. The genome alignment was used to highlight recombination hotspots using gubbins [66]. The generated phylotree and its corresponding data on recombinations was visualized by Phandango [67].

### 4.8. 16S rRNA Based Phylogeny

In silico PCR available on insilico.ehu.eus [68] was performed using the previously mentioned primers to extract the 16S rRNA sequences from the 16 reference genomes. The obtained sequences had an average length of 544 bp. The sequences were aligned using the Needle-Wunsch algorithm and an UPGMA tree was constructed on the BioNumerics v7.6.1 beta software (Applied Maths, Sint-Martens-Latem, Belgium).

### 4.9. Comparative Genome Analysis 

Full chromosome comparison through pairwise matching and alignment of N×N sequences was generated using BioNumerics v7.6.1 beta software (Applied Maths, Sint-Martens-Latem, Belgium). B196 was used as a template for later alignments. 

A circular genome representation of LAU_SINT was generated using BRIG through comparison with the other 16 genomes [69]. The average nucleotide identity of LAU_SINT and closely related isolates was estimated using the average nucleotide identity (ANI) calculator [70]. 

All SC (*n* = 13) and SA (*n* = 46) genomes available on NCBI, as of 20 October 2018, were retrieved and full chromosomal comparisons and wgSNPs-based phylogeny were constructed using BioNumerics v7.6.1 beta software (Applied Maths, Sint-Martens-Latem, Belgium). The dN/dS ratio was calculated for the detected SNPs. 

### 4.10. Pan-Genome Analysis

The LAU_SINT genome was annotated using Prokka version 1.13 [71] with a similarity cutoff e-value 10^−6^ and minimum contig size of 200 bp. The annotated GFF3 file was piped into Roary version 3.12.0 [24] choosing a minimum blastp identity of 95 and core gene prevalence in all (>99%) of the isolates. A maximum-likelihood phylogenetic tree based on the core genome alignment was constructed using RAxML [65] with the GRTGAMMA mode of nucleotide evolution and 1000 bootstrap iterations. The resulting phylogenetic tree with isolate metadata where available along with the pan genome fingerprints were visualized on Phandango V 1.1.0 [67]. Sixteen reference genomes were included in the comparison.

### 4.11. Data Availability 

The dataset generated during the current study is available in the DDBJ/ENA/GenBank repository under the accession number GCA_003284685.1.

## Figures and Tables

**Table 1:** List of isolates deposited on NCBI as SI with corresponding genome information and sample data. bp: base pairs; CDSs: number of coding sequences; NA: not available; Year: date of submission on NCBI; Ref.: reference.

#	Isolate	Size (bp)	GC%	RNA	CDS	Sample Information	Year	Country	Ref.	Accession #	Assembly Level
1	F0395	1,927,278	37.9	69	1958	NA	2011	USA	NA	GCA_000234015.1	Draft
2	F0413	1,921,347	37.6	69	1950	NA	2011	USA	NA	GCA_000234035.1	Draft
3	BA1	1,965,880	37.7	74	1971	Epidural abscess	2012	USA	Planet et al., 2013	GCA_000313655.1	Draft
4	SK54 = ATCC 27335	1,910,718	37.6	31	1883	NA	2012	USA	NA	GCA_000258445.1	Draft
5	JTH08	1,933,610	37.6	79	1890	NA	2012	Japan	NA	GCA_000306805.1	Complete
6	C270	1,960,728	37.6	72	1910	Human bronchopulmonary abscess	2013	Canada	Olson et al., 2013	GCA_000463385.1	Complete
7	B196	1,996,214	37.6	72	1923	Broncho-pulmonary abscess, septic arthritis, osteomyelitis, pyomyositis	2013	Canada	Olson et al., 2013	GCA_000463355.1	Complete
8	567_SINT	2,069,671	38.0	56	2073	NA	2015	USA	NA	GCA_001073405.1	Draft
9	631_SCON	1,968,691	37.8	57	1904	NA	2015	USA	NA	GCA_001073635.1	Draft
10	KCOM 1545	1,908,201	37.6	37	1870	Oral Cavity, enonotic infection; collected in 2005	2015	South Korea	NA	GCA_001296205.1	Complete
11	TYG1620	2,006,877	37.6	72	2007	Brain abscess in an infant	2016	Japan	Hasegawa et al., 2017	GCA_002356055.1	Complete
12	30309	1,956,646	37.5	46	1915	Abscess, pyogenic fluids of percutaneous drainage; collected in 2015	2018	China	NA	GCA_002879585.1	Draft
13	32811	1,970,384	37.7	48	1975	Abscess, pyogenic fluids of percutaneous drainage; collected in 2015	2018	China	NA	GCA_002879575.1	Draft
14	FDAARGOS_233	1,914,006	37.7	72	1868	Abscess in an 15-months female; collected in 2014	2018	USA	NA	GCA_002073355.2	Complete
15	LAU_SINT	1,950,264	37.7	54	1898	Meningitis and brain abscess in an 13-years old male	2018	Lebanon	This study	GCA_003284685.1	Draft
16	NCTC11324	1,932,951	37.7	72	1890	NA	2018	NA	NA	GCA_900475975.1	Complete
17	LC4	1,914,382	37.8	43	1871	Abscess, pyogenic fluids of percutaneous drainage; collected in 2015	2018	China	NA	GCA_002879755.1	Draft

## Supplementary Materials

The following are available online at http://www.mdpi.com/2076-0817/8/1/22/s1, Figure S1. wgSNPs-based tree UPGMA phylogenetic tree of all SAG isolates constructed using the Bionumerics software. Blue: *S. anginosus*; purple: *S. intermedius*; red: *S. constellatus*; Figure S2. wgSNPs-based tree UPGMA phylogenetic tree of the 17 SI genomes constructed using the Bionumerics software. NA: not available; Figure S3. 16S rRNA-based UPGMA phylogenetic tree constructed using the Bionumerics software. The sequences, having an average length of 544 bp, were aligned using the Needle-Wunsch algorithm; Figure S4. Phage content of the isolates; Figure S5. Pan-genome analysis of all 17 SI isolates; Figure S6. Comparison of the accessory genes in *S. intermedius* relating to drug resistance, toxins and transport systems; Table S1. Disc diffusion diameter cutoffs and results based on EUCAST January 2019 guidlines; Table S2. List of the regions showing mutations in LAU_SINT and SAG genomes and their corresponding dN/dS ratios; Table S3. The genomic islands carrying the four modules (I-IV) of T7SS in SI along with gene alignment resutls.
