# Supplementary figures and images for: Genome Mining and Comparative Analysis of Streptococcus intermedius Causing Brain Abscess in a Child
Source: Pathogens. 2019 Feb 13;8(1):22. doi: 10.3390/pathogens8010022 (PMC6471051; doi:10.3390/pathogens8010022)

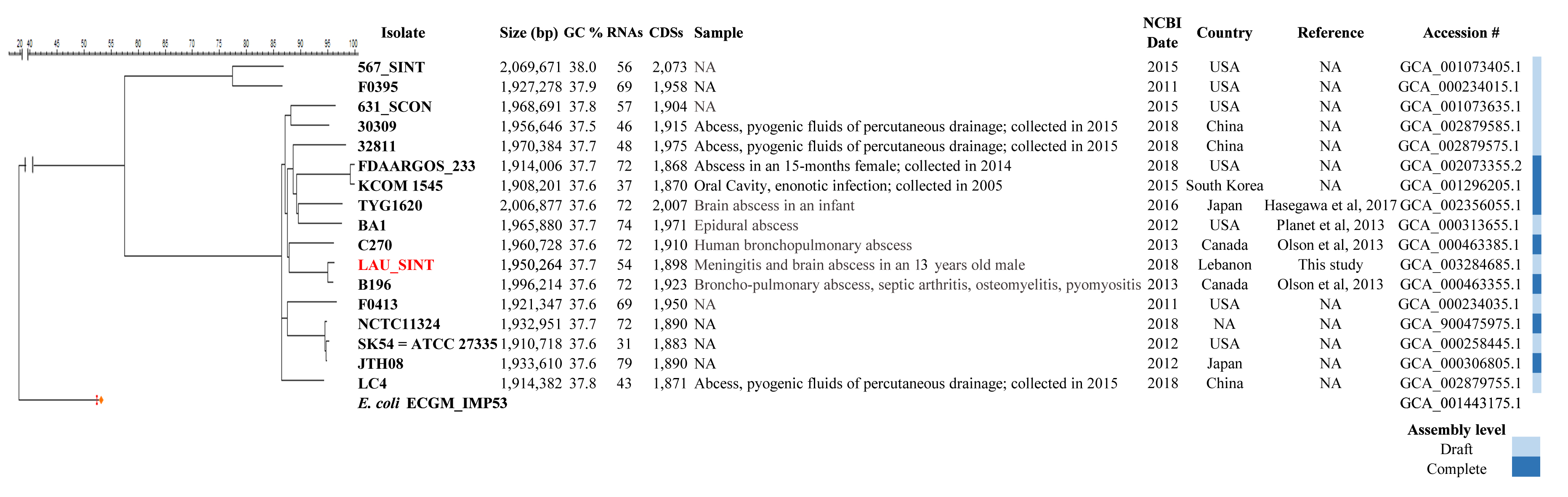

Supplement: Supplementary file 1 [file pathogens-08-00022-s001.zip › Pathogens_Suppl_Material_Jan9/Figure S2.jpg]

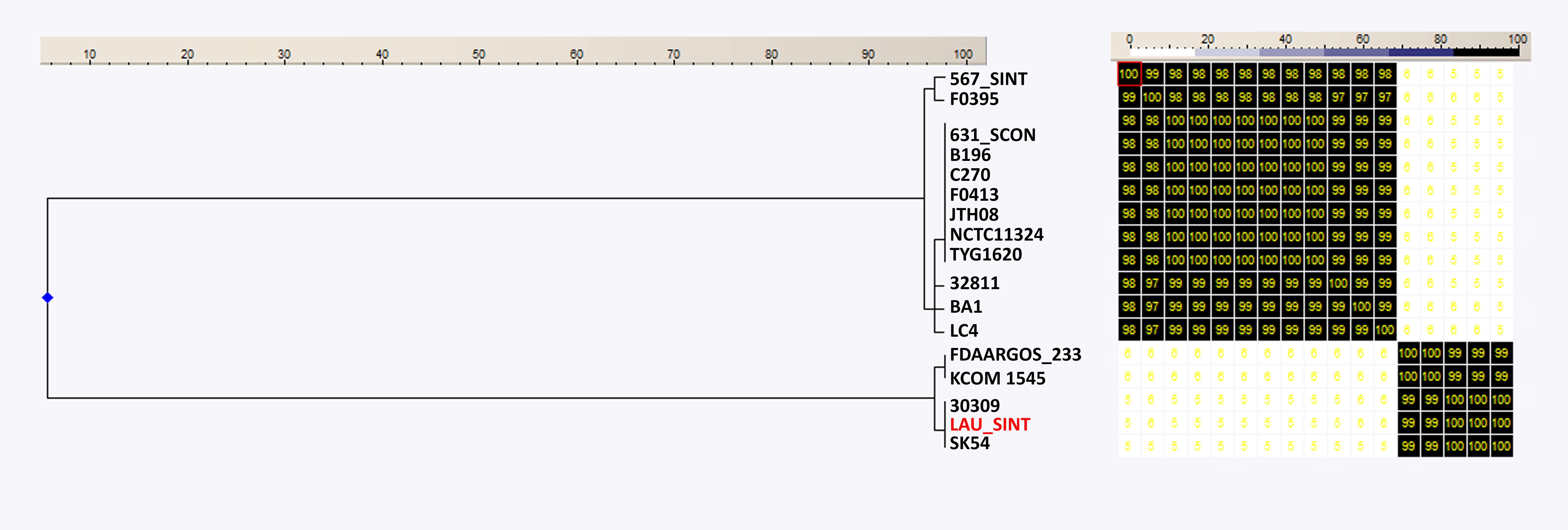

Supplement: Supplementary file 1 [file pathogens-08-00022-s001.zip › Pathogens_Suppl_Material_Jan9/FigureS3.jpg]

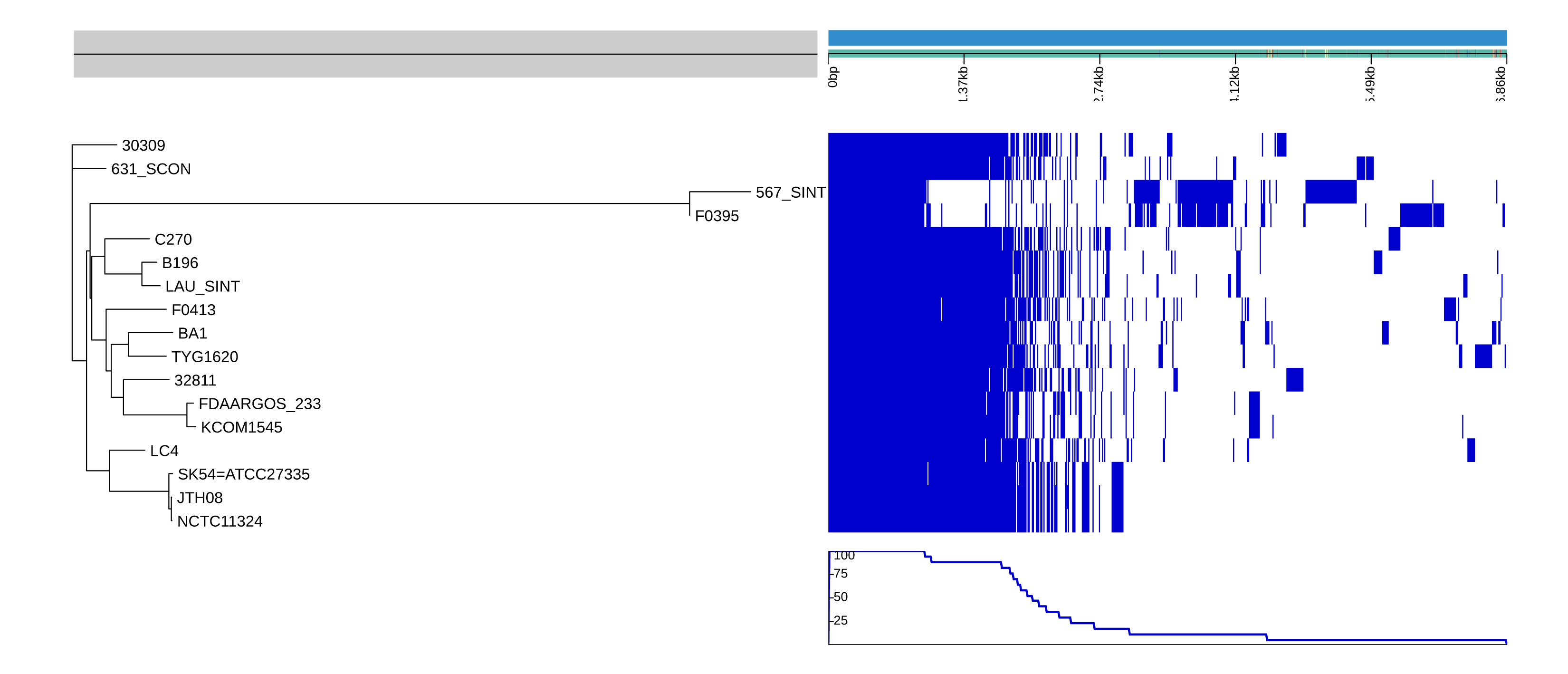

Supplement: Supplementary file 1 [file pathogens-08-00022-s001.zip › Pathogens_Suppl_Material_Jan9/Figure S5.jpg]

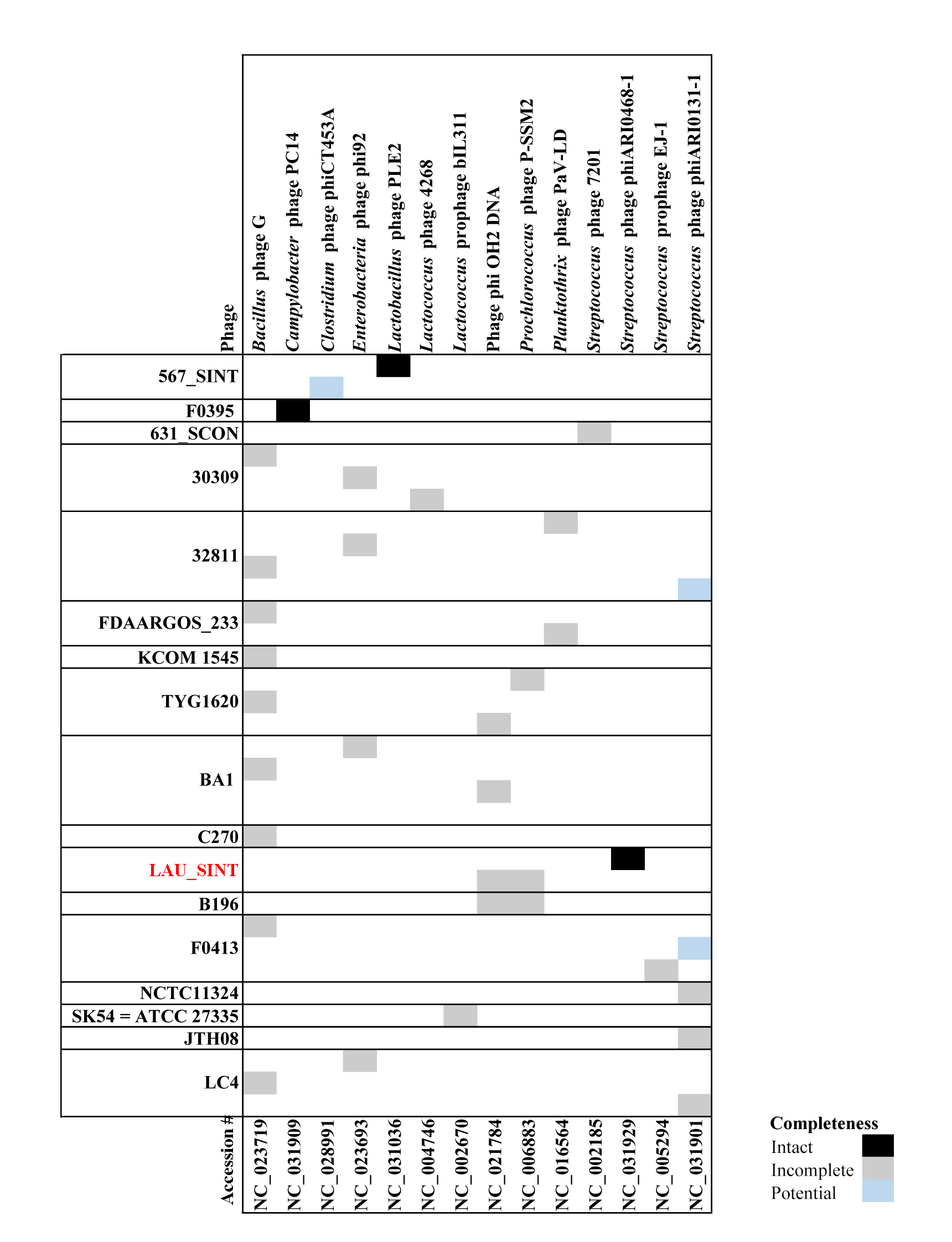

Supplement: Supplementary file 1 [file pathogens-08-00022-s001.zip › Pathogens_Suppl_Material_Jan9/Figure S4.jpg]

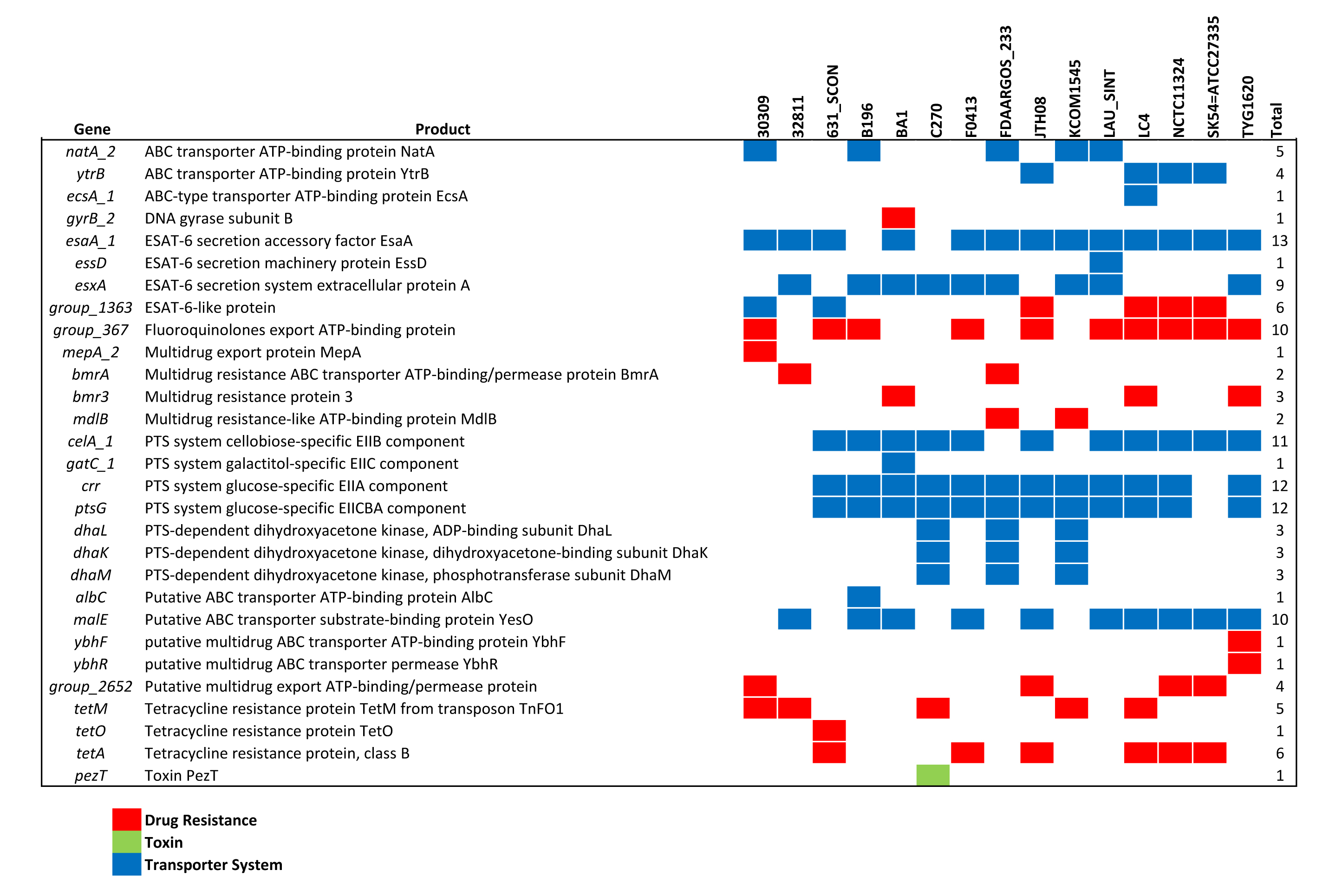

Supplement: Supplementary file 1 [file pathogens-08-00022-s001.zip › Pathogens_Suppl_Material_Jan9/Figure S6.jpg]
